# The Impact of Travel Distance on Cancer Stage at Diagnosis for Cancer: A Systematic Review

**DOI:** 10.3390/ijerph22040518

**Published:** 2025-03-28

**Authors:** Chaimaa Elattabi, Najoua Lamchabbek, Saber Boutayeb, Lahcen Belyamani, Inge Huybrechts, Elodie Faure, Mohamed Khalis

**Affiliations:** 1 Department of Public Health and Clinical Research, Mohammed VI Center for Research and Innovation, Rabat 10112, Morocco; nlamchabbek@um6ss.ma (N.L.); sboutayeb@cm6.ma (S.B.); belyamani@gmail.com (L.B.); mkhalis@um6ss.ma (M.K.); 2 Mohammed VI International School of Public Health, Mohammed VI University of Sciences and Health, Casablanca 82403, Morocco; 3 Faculty of Medicine, Mohammed VI University of Sciences and Health, Casablanca 43150, Morocco; 4 International Agency for Research on Cancer, World Health Organization, 69366 Lyon, France; huybrechtsi@iarc.who.int; 5 French Network for Nutrition and Cancer Research (Nacre Network), 78350 Jouy-en-Josas, France; 6 Université Paris-Saclay, UVSQ, Inserm, Gustave Roussy, CESP, 94805 Villejuif, France; elodie.faure@gustaveroussy.fr; 7 Higher Institute of Nursing Professions and Health Techniques, Rabat, Ministry of Health and Social Protection, Rabat 10000, Morocco

**Keywords:** cancer, tumor stage, travel distance, travel time, geographic access

## Abstract

Background: Geographic access to healthcare services can impact cancer outcomes. This paper reviews and updates the current evidence and gaps in the literature on the associations between travel distance and cancer stage. Methods: A search of electronic databases (PubMed, SpringerLink, and Science Direct) was conducted to identify studies published between 2015 and 2025. Studies examining the association between travel distance and cancer stage at diagnosis were included in this article. Results: From 19,197 studies, 11 articles met the inclusion criteria. In summary, four articles reported significant associations between travel distance/time and cancer stage, while six other articles did not report any association. Significant associations were observed in sub-Saharan Africa. In contrast, studies from Scotland, Canada, and the United States did not show significant relationships, while results from Japan varied, with papers showing either no significant impact of travel distance or indicating a correlation with advanced stages. Conclusions: This study suggests that longer travel distance is associated with advanced cancer stage in countries with healthcare access challenges and highlights the importance of healthcare accessibility in improving early cancer detection.

## 1. Introduction

Cancer remains one of the leading causes of morbidity and mortality worldwide, accounting for a significant global health burden.

Cancer is second leading cause of death in the world after coronary heart disease [1]. According to estimates from the World Health Organization (WHO), cancer is one of the leading causes of death before the age of 70 in 91 of 172 countries. In 2022, there were 20 million new cases and 9.7 million cancer-related deaths worldwide. Furthermore, the number of new cancer cases is projected to rise by about 50% over the next two decades. By 2040, the number of new cancer cases per year is expected to rise to 28.4 million and the number of cancer-related deaths to 16.4 million [2]. Despite advancements in prevention, early diagnosis, and treatment, disparities in cancer care persist, particularly among populations with limited access to healthcare facilities. Geographical factors, including travel distance to healthcare services, have been documented [3,4,5,6]. Many studies have reported that travel burden (measured as travel distance or travel time) can impact diagnosis and influence treatment choice [7,8], explaining that individuals living far from healthcare facilities often face delayed diagnosis and limited treatment options, which may contribute to poorer prognosis and advanced stages of cancer at diagnosis. However, the existing literature on this topic remains inconsistent, with studies varying in methodology, cancer type, and healthcare settings, making it challenging to draw comprehensive conclusions. In 2015, a review on the association between cancer outcomes and travel distance revealed that 10 out of 12 studies reported an association between advanced cancer stage and distance over 50 miles or 1 h or more in driving time [9]. In contrast, a systematic review examining the association between geographic access to mammography and breast cancer screening and stage at diagnosis reported that findings varied widely across studies, with no clear trends regarding the geographic level or the measurement tool (travel distance/travel time) [10]. In 2022, a systematic review focusing on geographic access and breast cancer outcomes found that the results varied depending on whether travel time or travel distance was used as the measure of accessibility [11]. While the previous review, published in 2015, focused on studies available up until that time, the present systematic review builds on the more recent literature, incorporating studies published after 2015. This allows to provide an updated and comprehensive overview of the relationship between travel distance or time and cancer stage at diagnosis to support evidence-based practice and identify gaps in the existing literature by specifically addressing the 10 most common cancers worldwide due to their global prevalence, high metastatic potential, significant contribution to cancer burden, and critical impact on public health and healthcare systems.

## 2. Materials and Methods

This systematic review was conducted according to the Preferred Reporting Items for Systematic Reviews and Meta-Analyses (PRISMA) statement by Moher et al. [12].

### 2.1. Protocol and Registration

The review protocol was registered in the database of the international prospective register of systematic reviews ‘PROSPERO’ under the number CRD42022332181, and it is available at the following link: https://www.crd.york.ac.uk/prospero/display_record.php?RecordID=332181 (accessed on 10 February 2025).

### 2.2. Data Sources and Search Strategy

Pre-selected Medical Subject Headings (MeSH) terms and text words were used to search PubMed, SpringerLink, and Science Direct using the following Medical Subject Headings and key terms that were combined by the Boolean operators ‘AND’, ‘OR’, and ‘Not’ to obtain several search equations according to the databases: ‘Breast cancer’, ‘prostate cancer’, ‘lung cancer’, ‘colorectum cancer’, ‘cervix cancer’, ‘stomach cancer’, ‘liver cancer’, ‘corpus uteri cancer’, ‘ovary cancer’, ‘thyroid cancer’, ‘distance’, ‘travel distance’, ‘travel time’,‘ travel burden’,‘ geographic access’,‘ distance to care’, ‘geospatial access’, ‘hospital’, ‘mammography’, ‘cancer screening service’, ‘health services’, ‘cancer diagnosis’, ‘diagnosis facility’, ‘cancer centers’, ‘cancer diagnosis Facility’, ‘tumor stage’, ‘tumor size’, ‘cancer stage’, ‘stage at diagnosis’, ‘cancer staging’, and ‘grade of malignancy’.

Articles were eligible for inclusion in this systematic review if they reported findings from primary research studies conducted among patients with 1 of the 10 most common cancers cited above: breast cancer, prostate cancer, lung cancer, colorectal cancer, cervix cancer, stomach cancer, liver cancer, corpus uteri cancer, ovary cancer, and thyroid cancer. The selected studies examined the geographic accessibility to cancer services, measured either in travel time or travel distance, and were published between January 2015 and January 2025. This timeframe was selected because the last review on cancer outcomes was conducted in 2015. There were no language restrictions, and there were no prior restrictions regarding the study design. Studies without abstracts or for which the full text was not available were excluded. Additionally, studies comparing rural and urban areas without measuring travel distance were excluded from the present review.

### 2.3. Study Selection and Data Collection Process

Zotero 6 reference manager software [13] was used to organize and detect duplicate references. We identified eligible articles by using the PRISMA flow diagram. The first and second authors independently screened all titles and abstracts identified through the search, and those clearly irrelevant to the topic were excluded. The full texts of all potentially eligible papers were retrieved and reviewed for inclusion in this review according to the inclusion criteria. All included studies were independently reviewed by two authors to confirm eligibility (EC and LN).

### 2.4. Data Extraction and Items

For the included studies, two authors (EC and NL) independently extracted information related to the characteristics of the study (title, authors, country, year of publication, study design, study period, research method, cancer type, and sample size), geographic access measurement (travel time/travel distance), and stage measurement (TNM classification, Figo stage, Duke stage).

Studies were examined, and the results were interpreted in relation to the country of study, allowing for comparisons of healthcare accessibility, infrastructure, and socioeconomic level. Additionally, results were also compared based on cancer type, which was classified into two categories: easy-to-diagnose cancer types, and hard-to-diagnose cancer types. This classification allows us to analyze the evidence based on the diagnostic challenges associated with each cancer type and to understand patient behavior and healthcare access. Easy-to-diagnose cancers, such as breast cancer and rectal cancer, are generally more accessible for early detection due to the availability of screening programs and more apparent symptoms, which can explain early medical consultation. Hard-to-diagnose cancers, such as stomach, pancreatic, lung, and ovarian cancer, often present non-specific symptoms or remain asymptomatic until they reach an advanced stage, making early diagnosis more challenging [14,15].

### 2.5. Quality Assessment

The quality of cohort studies was assessed independently by two authors (EC and LN) using the Newcastle–Ottawa Scale (NOS) quality assessment tool (Table S1). The NOS evaluates studies across three domains: selection, comparability, and outcome. Each domain was rated with a star system (four stars for selection, two stars for comparability, and three stars for outcome), and studies were classified as high-, moderate-, or low-quality based on their scores. The cross-sectional studies were evaluated using the AXIS tool (Table S2), which evaluates studies across various domains, such as clarity of objectives, appropriateness of the sampling method, measurement validity, data analysis, and ethical considerations.

## 3. Results

**Figure 1:**
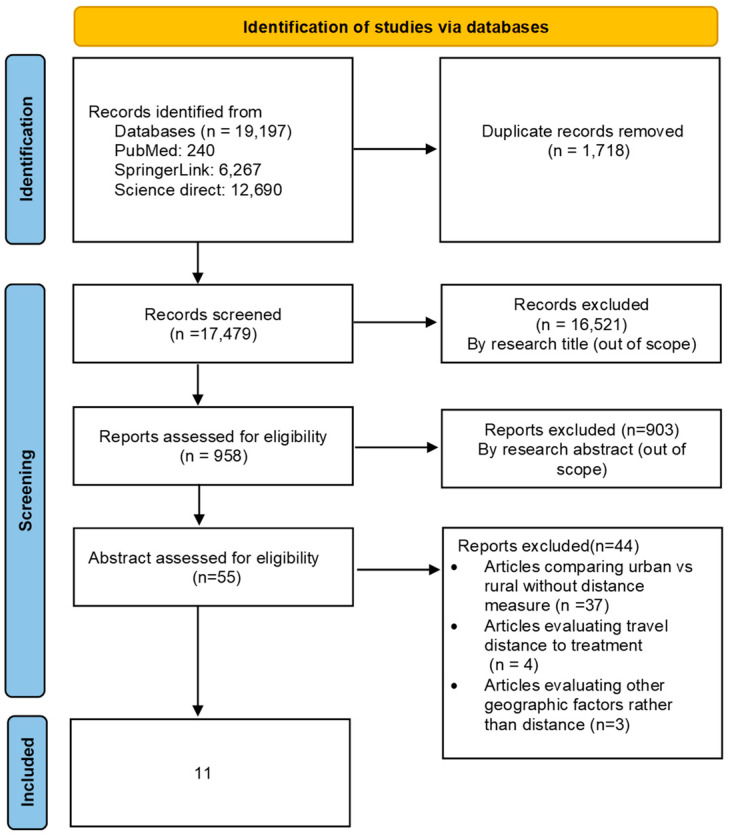
PRISMA flow diagram for studies’ selection.

A total of 19,197 studies were identified through the literature search. Following the removal of duplicate studies and those that fell outside of the scope of our review, only 55 studies remained for full-text assessment. Ultimately, 11 studies met the criteria for inclusion in our review (Figure 1).

The main characteristics of the included studies are summarized in Table 1. The articles selected were based on studies carried out in four developed countries and six developing countries: three in the USA [16,17,18], two in Japan [19,20], and one each in Canada [20], Ethiopia [21] Namibia, Nigeria, Uganda, and Zambia [22], Botswana [23], Denmark [24], and northeast Scotland [25]. The study publication dates ranged from 2015 to 2025. The sample sizes of the studies ranged from 390 to 256,663. Cancer types that were reported in the studies were colorectal cancer (*n* = 4), cervical cancer (*n* = 3), breast cancer (*n* = 3), lung cancer (*n* = 3), ovarian cancer (*n* = 2), stomach cancer (*n* = 1), and prostate cancer (*n* = 1) (Table 1).

In this systematic review, eight articles used the American Joint Committee on Cancer (AJCC) staging system for cancer stage. Two articles used the International Federation of Gynecology and Obstetrics (FIGO) staging system to determine cancer stage, and another article used Duke’s stage. Using geographic information systems (GIS), geographic access between patients’ place of residence and the nearest health facility was assessed by travel time [18,23,25] by straight line distance [19,22] or by the distance of the road network (driving distance) [20,24]. The 20 relationships between geographic access and cancer stage in the 11 papers reviewed are presented in Table 1. Overall, the statistical significance of the relationships was heterogeneous, with four significant relationships, six non-significant relationships, and one relationship that depended heavily on the cancer type. The results are presented based on the diagnosis difficulty and country of study.

### 3.1. Based on Diagnosed Cancer Type

For easy-to-diagnose cancer types, such as breast cancer, the evidence is controversial. For instance, Tesfaw et al. [21] found that in northwest Ethiopia, greater travel distance to healthcare facilities (more than 5 km) was associated with advanced-stage diagnosis of breast cancer (AOR = 3.2; 95% CI: 1.72, 5.29). Conversely, the results of Virgilsen et al. [24] indicate that for breast cancer, there is no significant association between distance to either hospitals or general practitioners (GPs) and the likelihood of being diagnosed at an advanced stage. When examining the distance to hospitals, the percentage of patients diagnosed at an advanced stage remained relatively stable, ranging from 44% for those living within 3 km to 42% for those living more than 62 km away (e.g., OR = 1.00, 95% CI: 0.94–1.08 for 3–20 km; OR = 0.96, 95% CI: 0.88–1.05 for >62 km) (NS). Similarly, for distance to a GP, the percentage of advanced stage diagnosis varied only slightly, from 43% in the closest category (≤0.5 km) to 44% in the farthest category (>9 km) (e.g., OR = 0.96, 95% CI: 0.91–1.02 for 0.5–2 km; OR = 1.06, 95% CI: 0.98–1.14 for >9 km), again with no significant *p*-values [24]. For hard-to-diagnose cancer types, such as stomach, pancreatic, lung, and ovarian cancer, the studies showed conflicting results. For example, Virgilsen et al., when examining the impact of travel distance on cancer stage for stomach cancer, showed that the geographic distance between the patient and the general practitioner was not significantly associated with an advanced-stage cancer diagnosis. Specifically, for patients living less than 0.5 km from the GP, the advanced-stage diagnosis rate was 81%, and the odds ratios (ORs) and adjusted odds ratios (AORs) for different distances (0.5–2 km, 2–9 km, >9 km) showed no significant variation in the odds of advanced-stage diagnosis, with ORs close to 1. In contrast, a significant association was observed between the distance to the hospital and the diagnosis of advanced-stage stomach cancer. Patients living within 0–3 km of the hospital had an advanced-stage diagnosis rate of 85%. Those living between 3 and 20 km had an adjusted OR of 0.79 (95% CI: 0.57–1.09), suggesting a reduction in the odds of advanced-stage diagnosis, while patients living more than 62 km away had an adjusted OR of 0.60 (95% CI: 0.42–0.86), indicating an even greater reduction in the odds of advanced-stage diagnosis [24].

Regarding lung cancer, Virgilsen et al. reported that the distance to the GP was not significantly associated with an advanced stage. Among those residing within 0.5 km, 81% were diagnosed at an advanced stage. Patients between 0.5 and 2 km from a GP had an OR of 0.92 (95% CI: 0.86–0.99) and an adjusted OR of 0.94 (95% CI: 0.88–1.00). Those residing 2 to 9 km away had an OR of 0.94 (95% CI: 0.88–1.00) and an adjusted OR of 0.95 (95% CI: 0.89–1.02), while those living over 9 km away had an OR of 0.84 (95% CI: 0.77–0.92) and an adjusted OR of 0.87 (95% CI: 0.79–0.95). However, distance to the hospital was significantly associated with an advanced-stage lung cancer diagnosis (*p* < 0.001). Those residing within 3 km had an advanced-stage diagnosis rate of 82%. Those between 3 and 20 km away had an OR of 0.94 (95% CI: 0.86–1.02) and an adjusted OR of 0.96 (95% CI: 0.88–1.05), while those between 20 and 62 km had an OR of 0.86 (95% CI: 0.79–0.93) and an adjusted OR of 0.87 (95% CI: 0.80–0.95). Patients living beyond 62 km had an advanced-stage diagnosis rate of 74%, with an OR of 0.61 (95% CI: 0.55–0.68) and an adjusted OR of 0.64 (95% CI: 0.57–0.72 [24] On the other hand, Takenaka et al. [20] concluded that lung cancer stage was similar across travel distance groups (<10 km, 10–30 km, and >30 km) (*p* = 0.07).

### 3.2. Based on Countries (Developing Countries vs. Developed Countries)

- Developing countries:

In sub-Saharan Africa, studies reported significant associations between travel distance and cancer stage. Togawa et al. [22] found that for every additional 50 km traveled, the odds of a late-stage diagnosis increase by 4% in sub-Saharan Africa. Similarly, in Ethiopia, Tesfaw et al. [21] reported that breast cancer patients traveling 5 km or more to the nearest healthcare facility were significantly more likely to be diagnosed at an advanced stage (AOR = 3.2; 95% CI: 1.72, 5.29). In Botswana, Klingner et al. [23] reported that cervical cancer patients with travel times of 3–5 h and over 5 h had higher odds of presenting with stage II disease compared to those with a travel time of 1 h (AOR = 2.00 and 2.19, respectively).

- Developed countries:

Studies conducted in northeast Scotland and Canada did not find an association between travel distance and cancer stage. Murage et al. [25], in their article on colorectal cancer in northeast Scotland, revealed that travel distance was not associated with Duke’s stage of cancer (*p* = 0.94). Similarly, in Canada, although Bosma et al. [26] did not consider stage I or IV, they reported no statistically significant differences in cancer stage based on travel distance (*p* = 0.680). Also, in the United States, Barrington et al. [16] found that cancer stage at diagnosis was similar across cervical cancer patients, suggesting that access to care might be more uniform for this patient population. Also, the studies by Petersen et al. [17] and Charlton et al. [18] reported that travel time was not associated with late stage for ovarian cancer or colorectal cancer, respectively. In Japan, Tanaka et al. [19] found that a distance ≥ 40 km from a general hospital or clinic was the longest distance of the stage at diagnosis (*p* < 0.001). However, Takenaka et al. [20] reported that patients living less than 10 km, between 10 and 30 km, and more than 30 km away had similar distributions of pathological stages, with 69%, 74%, and 63% being in stage I, respectively.

## 4. Discussion

We examined 11 papers on the association between travel distance and cancer stage at diagnosis. The studies used two measures to evaluate access to the nearest healthcare facility, including travel time and travel distance. Studies were conducted in developed countries (USA, Canada, Japan, and Denmark) and emerging countries (Ethiopia and sub-Saharan Africa). Sample sizes ranged from 220 to over 250,000, generally showing substantial statistical power and potential for reliable results.

Many articles considered rurality and socioeconomic status while accessing the impact of travel distance on cancer stage. For example, Bosma et al. classified patient’s residence into rural or urban based on distance to tertiary or regional cancer centers [26]. Murage et al. also considered rurality in their study [25]; when assessing the impact of travel time without interaction with rurality, no significant associations were found between travel time, rural–urban residence, and outcomes (alarm symptoms, emergency admissions, and cancer stage). When introducing an interaction term, the results showed that the effects of travel time varied by residence area [25].

The study by Togawa et al. examined the relationship with both rural residence and travel distance and considered socioeconomic status in their analysis [22]. Rurality was associated with delays in diagnosis, but this association weakened after adjusting for socioeconomic status. Conversely, longer travel distance remained an independent predictor of late stage at diagnosis even after controlling for socioeconomic status.

Even if our review found few articles that considered rurality and socioeconomic status, the interaction between these factors and travel distance cannot be ignored. Studies that did not account for these variables simultaneously may have underestimated the true impact of rurality and socioeconomic status or overestimated the role of travel distance in isolation. These findings highlight the need for a more nuanced approach that considers how economic disadvantage and residential barriers interact to influence cancer outcomes.

The results show that the association between travel distance and tumor stage is not homogeneous and that it varies significantly between cancer types and diagnosis difficulty, which is also supported by the existing literature across cancer types [16,24,25,27] These findings highlight the complexity of the relationship between travel distance and cancer stage, hypothesizing that factors other than distance alone might play significant roles in cancer diagnosis and staging.

It was hypothesized that patients with typically difficult-to-diagnose symptoms and a longer travel distance would be more strongly associated with advanced tumor stages. Conversely, for easier-to-diagnose cancer types, patients present with alarm symptoms, and tumor stage would not be influenced by travel distance, as their diagnostic process is clearer and thus less impacted by distance. In this review, two out of four easy-to-diagnose cancers and two out of five hard-to-diagnose cancers showed an association between late stage and travel distance, with some cancer types revealing an association with travel distance in certain studies and no association in others. Compared to the previous review by Ambroggi et al. [9], the results did not support the hypothesized pattern. Even among the cancer types included in the prior review—some of which fit within our classification of easy-to-diagnose and hard-to-diagnose cancers—the expected association between cancer type (breast cancer, rectum cancer, and lung cancer) and travel distance was not consistently observed.

In the present systematic review, the results reveal that longer travel distances to healthcare facilities were correlated with advanced cancer stages at diagnosis in Japan and African countries (Ethiopia, Botswana, Namibia, Nigeria, Uganda, and Zambia). And, results from north Scotland and Canada were not significant. However, studies included in the previous review conducted by Khan-Gates et al. on breast cancer were mixed and did not appear to be related to geographic area [10]. Conversely, the review conducted on cancer outcomes by Ambroggi et al. [9] reported that the results were almost all positive regardless of the country under study (Africa, USA, north Scotland, and Australia), highlighting that a publication bias could be present.

In the present review, the association between travel distance and cancer stage reflects complex interplays of healthcare infrastructure, socioeconomic conditions, and geographic barriers. Japan, despite its healthcare system and extensive access to medical services [28], faces challenges due to the geographic disparities in terms of specialized care facilities and mountainous terrains that requires long travel distances for patients living in rural areas, potentially leading to delays in cancer diagnosis [25] Sub-Saharan Africa also presents healthcare infrastructure limitations related to distribution of cancer treatment facilities and poor transportation networks [29]. These factors can explain timely access to healthcare services, resulting in delayed cancer diagnosis. Cultural factors and reliance on traditional medicine can also delay diagnosis and medical treatments. In Japan, for example, Kampo medicine, a traditional form of healing adapted from Chinese medicine, is widely used alongside conventional treatments [30,31]. Similarly, in sub-Saharan Africa, traditional medicine is very common in cultural practices and often considered as the first line of treatment due to factors like accessibility, cost, and mistrust of modern healthcare systems. Many patients consult traditional healers and use herbal treatments for extended periods before being referred to healthcare services [32]

Canada and Scotland both face significant challenges in delivering healthcare to rural and remote populations [33]. Rural patients in northern Canada have fewer healthcare facilities and lack healthcare professionals [33]. Similarly, in northern Scotland, patients often travel long distances to reach hospitals and specialists [34]. However, our studies from western Canada (Alberta) and northeast Scotland did not find any association between cancer stage and travel distance. This can be explained by the healthcare systems in both northern Scotland and Canada, which aim to provide universal healthcare access to all residents, regardless of their ability to pay. In Canada, the system known as Medicare is funded through taxes and provides universal coverage for medically necessary services, administered by each province and territory under the Canada Health Act [35]. Similarly, the National Health Service (NHS) of Scotland, funded through taxation, ensures that healthcare services are free at the point of use for all residents [36]. Also, both healthcare systems have implemented telehealth services to reduce the need for travel and provide remote consultations and follow-up care. In Canada, telehealth services are increasingly used to connect patients in remote areas with specialists in urban centers, thus reducing travel burdens and improving access to care. An example comes from Saskatchewan, Canada. In this province with a large rural population, telemedicine has led to over 17,000 appointments, saving patients more than 6 million kilometers in travel. This has reduced healthcare-related costs between rural and urban populations and reduced issues like waiting times, time away from work, urban commuting stress, and expenses related to travel, parking, and accommodation [37].

Scotland has also been investing in telehealth solutions to reach patients in remote areas, allowing them to consult with healthcare providers without needing to travel long distances [38]. For example, a randomized controlled trial [39], which evaluated telemedicine for multidisciplinary team meetings in breast cancer care in Scotland, revealed that telemedicine is a feasible and acceptable alternative to traditional face-to-face MDT meetings, and it could help bridge the gap in cancer care delivery [39]. Another study conducted among head and neck patients in Scotland found that telemedicine significantly reduced the need for patients to travel long distances for consultations [40].

The difference in findings could be influenced by many factors, including the characteristics of the study area. For example, Iowa is known for its extensive network of hospitals and healthcare facilities, which may reduce the impact of travel distance on cancer outcomes. On the other hand, Alabama may have different healthcare access issues, such as fewer specialized centers, potentially leading to different patterns in travel distance and cancer stage.

Moreover, results can differ even within the same cancer type. For example, the two studies on ovarian cancer [1,2] found different results, with one article showing an association between travel distance and cancer stage and another showing no association. This suggests that factors other than distance to specialized centers, such as socioeconomic status, referral patterns, and regional healthcare infrastructure, might influence cancer stage.

The quality assessment of the included studies indicated that most were of high methodological rigor. However, notable limitations exist, particularly regarding the measurement of travel distance, outcome definitions, and the variability in the adjustment variables used across studies. While some studies accounted for confounding factors, adjustments were often made for outcomes like survival rather than cancer stage at diagnosis. This inconsistency limits comparability and raises concerns about residual confounding. Additionally, the considerable heterogeneity in study designs, methodologies, and outcome measures prevented us from conducting a meta-analysis. The lack of standardized study designs, uniform distance definitions (straight line distance/road network distance/travel time), and harmonized adjustment variables made it difficult to synthesize findings quantitatively. A meta-analysis in the future could provide a more precise estimate of the association between travel distance and cancer stage at diagnosis, allowing for the quantification of effect sizes and improving the generalizability of results. However, achieving this requires future studies to adopt consistent methodologies, control for key confounding factors, and use standardized definitions of travel distance and cancer stage. Future research should also consider the influence of socioeconomic status and rurality when examining the impact of travel distance on cancer stage, as these factors may shape patients’ access to healthcare services. Lower-income individuals and those living in remote regions often face greater challenges in reaching medical facilities not only due to distance but also because of limited transportation options, higher travel costs, and time constraints. The mode of transportation—whether private vehicles, public transport, or reliance on ambulances—can significantly impact travel time and ultimately delay diagnosis. These factors can interact with travel distance, meaning that the burden of long distances may be longer for those with fewer financial resources or limited access to efficient transportation. To ensure greater transparency and accuracy, future studies should account for these interactions, allowing for a more precise understanding of the impact of travel distance on cancer stage.

## 5. Conclusions

This systematic review reveals that travel distance to healthcare facilities could influence cancer stage, with longer distances associated with advanced stages, particularly in regions with limited healthcare access. Future research should explore underlying factors contributing to these disparities and assess potential interventions to improve access to timely cancer diagnosis.

## Figures and Tables

**Table 1:** Characteristics of included studies.

Study Author, Publication Date	Country	Study Design	Study Period	Cancer Type	Sample Size	Age of Participants	Travel Distance Measurement	**Cancer Stage Measure**	**Results (OR (95% CI)/Correlation)**
Barrington et al. 2016 [16]	USA	Retrospective study	1999–2011	Cervical cancer	390	Median: 45.94	Distance was measured between patient’s residence and the Gynecologic Oncology clinic database and classified into two groups: -<100 miles -≥100 miles	FIGO classification	The two groups (<100 and ≥100 miles) had similar stages at diagnosis, with most having stage I lesions (61% vs. 71%, *p* = 0.377) (chi square test)
Tanaka et al. 2016 [19]	Japan	Retrospective study	2009–2011	Lung cancer	3,986	Not mentioned	Distance was measured in straight line distance from a patient’s place of residence to their hospital and classified into three groups: -<20 km -20–39.9 km -≥40 km	TNM classification	Using a Kruskal–Wallis test of distance and the stage at diagnosis, the proportion of ≥40 km from the general hospital and the clinic was the highest (*p* = 0.005)
Bosma et al. 2020 [26]	Canada	Retrospective study	2004–2015	Colon cancer (patients diagnosed with stage II/III colon cancer)	6163	Median: 71	Distance was measured from the patient’s residence to the nearest cancer center, and patients were classified into rural/urban areas based on their distance to the nearest cancer center: -Urban patients: Residing within 50 km of tertiary cancer centers -Suburban patients: Residing > 50 km of regional cancer centers -Rural patients: Residing > 50 km from any tertiary or regional cancer centers or community cancer centers	TNM classification	The groups had similar stages of disease. With regard to the proportions of patients diagnosed with stage III cancer, 49% (*n* = 1795) of urban patients, 50% (*n* = 882) of rural patients, and 48% (*n* = 331) of suburban patients were diagnosed (*p* = 0.680)
Petersen et al. 2021 [17]	USA	Retrospective study	2010–2015	Ovarian cancer	220	Median: 60.6	Distance was measured from the patient’s residence to the University of Kansas Cancer Center and classified into two groups: -<10 miles -≥10 miles	TNM classification	The two strata had similar cancer stages at diagnosis (*p* = 0.4694)
Tesfaw et al. 2021 [21]	Ethiopia	Cross-sectional study	1 September 2019–30 April 2020	Breast cancer	Not mentioned	Median:40	Distance was measured from the patients’ home to nearby healthcare facilities and classified into two groups: -<5 km -≥5 km	TNM classification	Travel distance to a nearby healthcare facility ≥5 km was associated with advanced-stage diagnosis of patients with breast cancer (AOR = 3.2; 95% CI: (1.72, 5.29))
Togawa et al. 2020 [22]	Sub-Saharan Africa (Namibia, Nigeria, Uganda, and Zambia)	Cohort study	In Namibia, Uganda, and Nigeria: September-December 2014–April 2017 In Zambia: May 2016–September 2017	Breast cancer	1541	Median: 50	Distance was measured in straight line distance from residential home to the first healthcare provider visited and sorted in ascending order and divided into four equal groups (quartiles)	TNM classification	Travel distance to cancer diagnostic or treatment facilities was associated with both delay in diagnosis and more advanced stage at diagnosis (OR per 50 km Increment: OR = 1.04)
Takenaka et al. 2016 [20]	Japan	Retrospective study	2006–2011	Non-small-cell lung cancer	607	Classified into three groups (<65 years, 65–74 years, and ≥75 years)	Distance was measured based on driving distance between the hospital and the patient’s residence and classified into three groups: -<10 km -10–30 km ->30 km	TNM classification	Patients living less than 10 km away, between 10 and 30 km away, and more than 30 km away had similar distributions of pathological stages, with 69%, 74%, and 63% being in stage I (*p* = 0.07)
Murage et al. 2017 [25]	Northeast Scotland	Cross-sectional study	1997–1998	Colorectal cancer	926	83.1% were over 60	Distance was measured based on estimated travel time (min) from the patients’ home postcode to the postcode of their general practitioner registration at diagnosis	Duke stage	No significant relationship between travel time and Duke’s stage (OR = 0.91) (95% CI: 0.78 to 1.07)
Charlton et al. 2016 [18]	USA	Retrospective study	2002–2009	Colorectal cancer	5792	Between 65 and 84	Distance was measured based on estimated travel time (min) from the patient‘s residential postal code to the postal code of the nearest colonoscopy provider	TNM classification	Travel time to colonoscopy was not associated with late stage. (Log rank test, *p* = 0.11). No significant differences were observed in travel times after stratifying by urban vs. rural residence
Virgilsen et al. 2016 [24]	Denmark	Retrospective study	2005–2016	Breast, testis, esophageal, colon; cervix, prostate, stomach, pancreatic, lung, ovarian cancer	256,663	Median: 50.8	Distance was measured based on driving distance from the patient’s residence to the general practitioner/hospital	TNM classification	1.Easy-to-diagnose cancers Rectal cancer: Higher odds of advanced-stage diagnosis for patients >62 km from hospitals (AOR: 1.32; 95% CI: 1.10–1.59; *p* = 0.002). Testis cancer: Strong association with distance, AOR: 2.55 (1.62–4.01) for >62 km (*p* < 0.001) 2. Intermediate-to-diagnose cancers Esophageal cancer: No significant association (NS) Colon cancer: No significant association (NS) Cervical cancer: Increased risk at >62 km (AOR: 1.15; 95% CI: 0.82–1.61; *p* = 0.021) Prostate cancer: Protective effect with increasing distance (lower OR at greater distances, *p* = 0.021) 3. Hard-to-diagnose cancers Stomach cancer: Lower odds of advanced stage with increased distance (AOR: 0.60 for >62 km, *p* = 0.006) Pancreatic cancer: Significant Lung cancer: Reduced odds of advanced stage at greater distances (AOR: 0.64 for >62 km, *p* < 0.001)
Klingner et al. 2022 [23]	Botswana	Retrospective study	Between 2015 and 2020	Cervical cancer	959	50.7	Distance was measured based on estimated ravel time from a patient’s residential village to the multidisciplinary team clinic and classified into four groups: -<1 h -1 h–3 h -3 h–5 h ->5 h	FIGO classification	Using a reference group of stage I disease and a travel time of 1 h, the odds of presenting with stage II increased for patients traveling 3–5 h (adjusted odds ratio [OR], 2.00; 95% CI, 1.14 to 3.52) and 5 h (OR, 2.19; 95% CI, 1.15 to 4.19). There were no significant associations for stage III. For stage IV disease, the odds were increased for patients traveling 3–5 h (OR, 2.93; 95% CI, 1.26 to 6.79) and 5 h (adjusted OR, 4.05; 95% CI, 1.62 to 10.10)

## Data Availability

No new data were created in this study.

## Supplementary Materials

The following supporting information can be downloaded at: https://www.mdpi.com/article/10.3390/ijerph22040518/s1, Table S1: Quality Assessment Using Newcastle-Ottawa Scale (NOS) for Cohort Studies; Table S2: Quality Assessment Using AXIS Tool for Cross-Sectional Studies.

## Abbreviations

The following abbreviations are used in this manuscript:

WHO	World Health Organization
USA	United States of America
FIGO	International Federation of Gynecology and Obstetrics
