# Supplementary material for: The Impact of Travel Distance on Cancer Stage at Diagnosis for Cancer: A Systematic Review
Source: Int J Environ Res Public Health. 2025 Mar 28;22(4):518. doi: 10.3390/ijerph22040518 (PMC12027156; doi:10.3390/ijerph22040518)
Supplement: Supplementary file 1 [file ijerph-22-00518-s001.zip › ijerph-3447059-supplementary.pdf]

Table S1: Quality Assessment Using Newcastle-Ottawa Scale (NOS) for Cohort Studies

Article Title	*Selection*	*Comparability*	*Outcome*
**Influence of Distance from Home to Hospital on Survival Among Lung Cancer Patients**	★★★	★★	★
**Disparities in Ovarian Cancer Survival at the Only NCI-Designated Cancer Center in Kansas**	★★★	★★	★
**Geospatial Barriers to Healthcare Access for Breast Cancer Diagnosis in Sub-Saharan African Settings: The African Breast Cancer—Disparities in Outcomes Cohort Study**	★★★	★★	★
**Influence of The Distance Between Home and The Hospital on Patients with Surgically Resected Non-Small-Cell Lung Cancer: The Google Maps Distance Calculator**	★★★	★★	★★
**Is Travel Time Associated with Late-Stage Colorectal Cancer Among Medicare Beneficiaries in Iowa?**	★★★	★★	★
**Travel Distance to Cancer-Diagnostic Facilities and Tumor Stage**	★★★	★★	★★
**Distance to Multidisciplinary Team Clinic in Gaborone, Botswana, and Stage at Cervical Cancer Presentation for Women Living with and Without HIV**	★★★	★★	★
**Distance from A Comprehensive Cancer Center: A Proxy for Poor Cervical Cancer Outcomes?**	★★★	★★	★
**Impact of Travel Time and Rurality on Presentation and Outcomes of Symptomatic Colorectal Cancer: A Cross-Sectional Cohort Study in Primary Care**	★★★	★★	★
**Characterizing Urban-Rural Differences in Colon Cancer Outcomes: A Population-Based Analysis Based on Travel Distance to Cancer Center**	★★★	★★	★

Table S2: Quality Assessment Using AXIS Tool for Cross-Sectional Studies

Article Title	***Study Design***	***Clarity of Objectives***	***Sampling Method***	***Measurement Validity***	***Data Analysis***	***Ethical Considerations***	***Total Quality***
**Factors Associated with Advanced-Stage Diagnosis of Breast Cancer In North-west Ethiopia: A Cross-Sectional Study**	Cross-Sectional Study	Clear	Appropriate	Not specified	Appropriate	Mentioned	High

Data sources	***Search terms/Equations***
PubMed	“Travel distance” AND “cancer”
	((("Travel/diagnosis"[Mesh] OR "Travel/epidemiology"[Mesh] OR "Travel/prevention and control"[Mesh])) AND "Health Facilities"[Mesh]) AND "Neoplasms"[Mesh]
	("Cancer Care Facilities"[Mesh]) AND "Neoplasm Staging"[Mesh]
	“Rural” and urban areas” AND “cancer “AND “cancer stage”<br>“Rural and urban areas” AND “cancer” AND “tumour stage”
	“Travel distance” AND “cancer outcomes”
	((((“Breast cancer”OR”prostate cancer” OR “lung cancer” OR “colorectum cancer” OR “cervix cancer” OR “stomach cancer” OR “liver cancer” OR “corpus uteri cancer” OR “ovary cancer” OR “thyroid cancer”) AND (“distance” OR “travel distance” OR “travel time” OR “travel burden” OR “geographical access” OR “distance to care” OR “geospatial access”)) AND (“hospital” OR “mammography” OR “cancer screening service” OR “health services” OR “cancer diagnosis” OR “radiation facility” OR “diagnosis facility” OR “cancer centers” ,OR “cancer diagnosis facility” OR “treatment facility”)) AND (“tumour stage” OR “tumour size” OR “cancer stage” OR “stage at diagnosis” OR “cancer staging” OR “grade of malignancy”))
	"Breast cancer" AND "distance" AND "tumor stage" AND "hospital"
	"Prostate cancer" AND "distance" AND "tumor stage" AND "clinic"
	"Lung cancer" AND "distance" AND "tumor stage" AND "hospital"
	"Colorectal cancer" AND "distance" AND "tumor stage" AND "hospital"
	"Cervix cancer" AND "distance" AND "tumor stage" AND "clinic"
	"Stomach cancer" AND "distance" AND "tumor stage" AND "hospital"
	"Liver cancer" AND "distance" AND "tumor stage" AND "hospital"

	"Corpus uteri cancer" AND "distance" AND "tumor stage" AND "hospital"
	"Ovary cancer" AND "distance" AND "tumor stage" AND "hospital"
	"Thyroid cancer" AND "distance" AND "tumor stage" AND "hospital"
	"Breast cancer" AND "distance" AND "tumor stage" AND "treatment facility"
	"Prostate cancer" AND "distance" AND "tumor stage" AND "cancer diagnosis facility"
	"Lung cancer" AND "distance" AND "tumor stage" AND "treatment facility"
	"Colorectal cancer" AND "distance" AND "tumor stage" AND "treatment facility"
	"Cervix cancer" AND "distance" AND "tumor stage" AND "cancer screening service"
	"Stomach cancer" AND "distance" AND "tumor stage" AND "treatment facility"
	"Liver cancer" AND "distance" AND "tumor stage" AND "cancer diagnosis facility"
	"Corpus uteri cancer" AND "distance" AND "tumor stage" AND "treatment facility"
	"Ovary cancer" AND "distance" AND "tumor stage" AND "cancer screening service"
	"Thyroid cancer" AND "distance" AND "tumor stage" AND "treatment facility"
	"Breast cancer" AND "distance" AND "stage" AND "hospital"
	"Prostate cancer" AND "distance" AND "stage" AND " cancer screening service "
	"Lung cancer" AND "distance" AND "stage" AND "hospital"
	"Colorectal cancer" AND "distance" AND "stage" AND "hospital"
	"Cervix cancer" AND "distance" AND "stage" AND "health care providor"
	"Stomach cancer" AND "distance" AND "stage" AND "hospital"
	"Liver cancer" AND "distance" AND "stage" AND "clinic"
	"Corpus uteri cancer" AND "distance" AND "stage" AND "hospital"
	"Ovary cancer" AND "distance" AND "stage" AND "geographic facility"
	"Thyroid cancer" AND "distance" AND "stage" AND "hospital"
	"Breast cancer" AND "distance" AND "stage" AND "treatment facility"
	"Prostate cancer" AND "distance" AND "stage" AND "cancer diagnosis facility"
	"Lung cancer" AND "distance" AND "stage" AND "treatment facility"
	"Colorectal cancer" AND "distance" AND "stage" AND "treatment facility"
	"Cervix cancer" AND "distance" AND "stage" AND "cancer screening service"
	"Stomach cancer" AND "distance" AND "stage" AND "treatment facility"
	"Liver cancer" AND "distance" AND "stage" AND "cancer diagnosis facility"
	"Corpus uteri cancer" AND "distance" AND "stage" AND "treatment facility"
	"Ovary cancer" AND "distance" AND "stage" AND "cancer screening service"
	"Thyroid cancer" AND "distance" AND "stage" AND "treatment facility"
	"Breast cancer" AND "distance" AND "advanced stage " AND "hospital"
	"Prostate cancer" AND "distance" AND "advanced stage" AND "geographic access"
	"Lung cancer" AND "distance" AND "advanced stage" AND "hospital"

Springer Link and science direct	"Colorectal cancer" AND "distance" AND "malignancy" AND "hospital"
	"Cervix cancer" AND "distance" AND "malignancy" AND "geospatial access"
	"Breast cancer" AND "distance" AND "hospital"
	"Prostate cancer" AND "travel distance" AND "mammography"
	"Lung cancer" AND "travel time" AND "cancer screening service"
	"Colorectal cancer" AND "travel burden" AND "health services"
	"Cervix cancer" AND "geographical access" AND "cancer diagnosis"
	"Stomach cancer" AND "distance to care" AND "radiation facility"
	"Liver cancer" AND "geospatial access" AND "diagnosis facility"
	"Corpus uteri cancer" AND "hospital" AND "cancer centers"
	"Ovary cancer" AND "mammography" AND "cancer diagnosis facility"
	"Thyroid cancer" AND "treatment facility" AND "tumour stage"
	"Breast cancer" AND "geographical access" AND "cancer staging"
	"Prostate cancer" AND "distance to care" AND "grade of malignancy"
	"Lung cancer" AND "distance" AND "stage at diagnosis"
	"Colorectal cancer" AND "travel distance" AND "tumour size"
	"Cervix cancer" AND "travel time" AND "cancer stage"
	"Stomach cancer" AND "travel burden" AND "cancer centers"
	"Liver cancer" AND "geographical access" AND "tumour stage"
	"Corpus uteri cancer" AND "distance to care" AND "grade of malignancy"
	"Ovary cancer" AND "geospatial access" AND "tumour size"
	"Thyroid cancer" AND "hospital" AND "cancer staging"
